# Central Venous-to-Arterial CO_2_ Gap Is a Useful Parameter in Monitoring Hypovolemia-Caused Altered Oxygen Balance: Animal Study

**DOI:** 10.1155/2013/583598

**Published:** 2013-08-29

**Authors:** Szilvia Kocsi, Gabor Demeter, Daniel Erces, Eniko Nagy, Jozsef Kaszaki, Zsolt Molnar

**Affiliations:** ^1^Department of Anaesthesiology and Intensive Therapy, University of Szeged, Semmelweis Utca 6., Szeged 6725, Hungary; ^2^Department of Anaesthesiology and Intensive Therapy, MH Honved Hospital, Róbert Károly Körút 44., Budapest 1134, Hungary; ^3^Institute of Surgical Research, University of Szeged, Pécsi Utca 6., Szeged 6720, Hungary

## Abstract

Monitoring hypovolemia is an everyday challenge in critical care, with no consensus on the best indicator or what is the clinically relevant level of hypovolemia. The aim of this experiment was to determine how central venous oxygen saturation (ScvO_2_) and central venous-to-arterial carbon dioxide difference (CO_2_ gap) reflect hypovolemia-caused changes in the balance of oxygen delivery and consumption. Anesthetized, ventilated Vietnamese minipigs (*n* = 10) were given a bolus followed by a continuous infusion of furosemide. At baseline and then in five stages hemodynamic, microcirculatory measurements and blood gas analysis were performed. Oxygen extraction increased significantly, which was accompanied by a significant drop in ScvO_2_ and a significant increase in CO_2_ gap. There was a significant negative correlation between oxygen extraction and ScvO_2_ and significant positive correlation between oxygen extraction and CO_2_ gap. Taking ScvO_2_ < 73% and CO_2_ gap >6 mmHg values together to predict an oxygen extraction >30%, the positive predictive value is 100%; negative predicted value is 72%. Microcirculatory parameters, capillary perfusion rate and red blood cell velocity, decreased significantly over time. Similar changes were not observed in the sham group. Our data suggest that ScvO_2_ < 73% and CO_2_ gap >6 mmHg can be complementary tools in detecting hypovolemia-caused imbalance of oxygen extraction.

## 1. Introduction

Diagnosing hypovolemia is an everyday challenge in critical care. Clinicians utilize a large array of tools from simple clinical signs to invasive hemodynamic measurements, but a universally accepted gold standard remains elusive [1]. Although diagnosis may prove difficult, early recognition of hypovolemia is of utmost importance. By the time macrohemodynamic changes manifest, the microcirculation may already be damaged [2]. Furthermore, fluid therapy is a double-edged sword: on the one hand fluid resuscitation can save lives, but on the other hand a cumulative positive fluid balance is an independent factor for mortality [3, 4]. Deciding on the level of monitoring (noninvasive, “less” invasive, invasive) and which parameter to monitor in order to keep the critically ill patient normovolemic remains uncertain.

Central venous oxygen saturation (ScvO_2_), an easily obtained parameter via the central venous catheter already *in situ* in most critically ill patients, is often used as a marker of the balance between oxygen delivery (DO_2_) and consumption (VO_2_). The main factors, which influence ScvO_2_, are hemoglobin (Hb), arterial oxygen saturation (SaO_2_), cardiac output (CO), and VO_2_. Theoretically if Hb, SaO_2_, and VO_2_ are kept constant, the value of ScvO_2_ should reflect the change in CO. Recent studies have translated theory into practice and demonstrated that ScvO_2_ may be a good marker for assessing fluid responsiveness [5, 6].

The normal value of ScvO_2_ varies between 73 and 82%. It is slightly higher than mixed-venous oxygen saturation (SvO_2_) and is considered a reasonable surrogate marker in the clinical setting [7]. 

Changes in ScvO_2_ reflect systemic oxygen uptake but may be falsely positive (>70%) in regional hypoxia [8]. Under these conditions the central venous-to-arterial CO_2_ difference (CO_2_ gap) has been proposed as an alternative [8–10]. The physiological value of CO_2_ gap is <5 mmHg, but this may be higher in low-flow states [8, 9]. However, it remains unclear how and whether the CO_2_ gap changes in hypovolemia.

Therefore, the aim of our hypovolemic animal model was to investigate the association between ScvO_2_, CO_2_ gap, microcirculatory blood flow and hypovolemia-caused altered VO_2_/DO_2_. 

## 2. Methods

The study protocol was approved by the local Ethics Committee and the Institutional Animal Care and Use Committee at the University of Szeged, and the study was conducted in the research laboratory of the Institute of Surgical Research in a manner that does not inflict unnecessary pain or discomfort upon the animal.

### 2.1. Animals and Instrumentation

Vietnamese minipigs (*n* = 15) weighing 28 ± 4 kg underwent a 24 hr fast preoperatively but with free access to water. Anesthesia was induced by intramuscular injection of a mixture of ketamine (20 mg/kg) and xylazine (2 mg/kg) and maintained with a continuous infusion of propofol (6 mg/kg/hr i.v.), while analgesia was maintained with nalbuphine (0.1 mg/kg). A tracheal tube was inserted and the animals' lungs were ventilated mechanically. The tidal volume was set at 10 mL/kg, and the respiratory rate was adjusted to maintain the end-tidal carbon dioxide and partial pressure of arterial carbon dioxide in the range of 35–45 mmHg and the arterial pH between 7.35 and 7.45. The adequacy of the depth of anesthesia was assessed by monitoring the jaw tone. After induction of anesthesia, the right jugular vein and the right femoral artery and vein were dissected and catheterized. Tonometric probes and catheters were placed simultaneously into the stomach and the small bowel. A suprapubic urinary catheter was also inserted to monitor urine output. Animals were kept warm (35 ± 1°C) by an external warming device.

For invasive hemodynamic monitoring, a transpulmonary thermodilution catheter (PiCCO, PULSION Medical Systems SE, Munich, Germany) was placed in the femoral artery, and a pulmonary artery catheter (PV2057 VoLEF Catheter, PULSION Medical Systems SE, Munich, Germany) was placed in the femoral vein. The latter was also used to draw mixed venous blood gas samples from which the VO_2_ was calculated. The femoral artery served as the site for arterial blood gas sampling and the central venous line was used for taking central venous blood gas samples and for the injection of cold saline boluses for the thermodilution measurements.

For continuous noninvasive visualization of the microcirculation in the sublingual region an intravital orthogonal polarization spectral (OPS) imaging technique (Cytoscan A/R, Cytometrics, Philadelphia, PA, USA) was used [2, 11]. A 10x objective was introduced onto the sublingual serosa, and microscopic images were recorded with an S-VHS video recorder (Panasonic AG-TL 700, Osaka, Japan).

For the tonometry special probes (Tonosoft Medical-Technical and R&G Ltd.) were used and monitoring was performed with a Sidestream Microcap Handheld Capnograph (Oridion Medical Ltd., Jerusalem, Israel) instrument [12].

To assess further biochemical changes in the microcirculation, plasma big-endothelin-1 (BigET) levels were determined. BigET is a 38 amino acid containing protein, the precursor of endothelin-1, which becomes elevated in tissue hypoxia [13].

### 2.2. Hemodynamic Measurements

Cardiac output (CO), global end-diastolic volume index (GEDI), stroke volume (SV), heart rate (HR), and mean arterial pressure (MAP) were measured by transpulmonary thermodilution and pulse contour analysis at baseline and at the end of each interval. Detailed description of transpulmonary thermodilution and pulse contour analysis is provided elsewhere [14, 15]. All hemodynamic parameters were indexed for body surface area or bodyweight. The average of three measurements following 10 mL bolus injections of ice-cold 0.9% saline was recorded. Central venous pressure (CVP) was measured via the central venous catheter at the same times as the other hemodynamic variables.

Arterial, central venous, and mixed venous blood gas samples (Cobas b 221, Roche Ltd., Basel, Switzerland) were drawn and analyzed by cooximetry simultaneously at baseline and at the end of each cycle.

### 2.3. Monitoring the Microcirculation

 Microcirculatory evaluation of the sublingual region was performed offline by frame-to-frame analysis of the videotaped images. Capillary red blood cell velocity (RBCV) and capillary perfusion rate (CPR) were determined in three separate fields using a computer-assisted image analysis system (IVM Pictron, Budapest, Hungary). All OPS measurements were performed by one investigator.

Gastric and small bowel changes in partial pressure of carbon dioxide (ΔPCO_2_) were calculated by subtracting tonometric PCO_2_ from arterial PCO_2_ (gastric-arterial: Ga-PCO_2_; bowel-arterial: Ba-PCO_2_) [12].

For measurements of BigET, blood samples of 2 mL were drawn from the jugular vein into chilled polypropylene tubes containing EDTA (1 mg/mL). The samples were centrifuged at 1200 g for 10 min at 4°C. The plasma samples were then collected and stored at −70°C until assay.

### 2.4. Experimental Protocol

At baseline (*T*_0_) hemodynamic, microcirculatory and blood gas parameters were recorded. Hypovolemia was induced via a bolus followed by a continuous infusion of furosemide (5 mg/kg and 5 mg/kg/2 h, resp.) in a group of 10 animals—hypovolemic group (HG). After the administration of bolus furosemide measurements were recorded in five stages with 20-minute interval between each measurement (*T*_1_–*T*_5_). When the preload parameter (GEDI) decreased by >20% its baseline value, OPS imaging and BigET sampling were performed, which were repeated only at the end of the experiment. There were 5 anaesthetised, ventilated animals in the sham group (SG), who did not receive any furosemide, but maintenance infusion of lactated Ringer (4 mL/kg/h) and hemodynamic, microcirculatory and blood gas parameters were recorded in the same fashion as described previously. At the end of the experiment all animals were humanely euthanized. 

### 2.5. Data Analysis and Statistics

Data are reported as means ± standard deviations unless indicated otherwise. For testing normal distribution the Kolmogorov-Smirnov test was used. Changes in all parameters throughout the experiment were tested by repeated measures analysis of variance (RM ANOVA), and the number of degrees of freedom was adjusted to Greenhouse-Geisser epsilon when needed. Mann-Whitney *U*-test with Bonferroni correction was used for between-groups analysis. For pairwise comparisons Pearson's correlation was used. To evaluate the performance of ScvO_2_, CO_2_ gap and microcirculatory parameters in detecting altered oxygen extraction with a threshold of 30%, receiver operating characteristics (ROC) curve analysis was performed, and sensitivity, specificity, positive predictive (PPV), and negative predictive values (NPV) were also determined. Post hoc calculation showed a power of 83% with an effect of 36% decrease in GEDI for a sample size of 10 and *α* = 0.05. For statistical analysis SPSS version 18.0 for Windows (SPSS, Chicago, IL, USA) was used and *p* < .05 was considered statistically significant.

## 3. Results

### 3.1. Hemodynamic Effects of Hypovolemia

Urine output in the hypovolemic group following the bolus and the onset of infusion was 176 ± 160 mL at *T*_1_, which increased to 647 ± 231 mL at *T*_5_. In contrast, in the sham group, urine output was 74 ± 74 mL at *T*_1_ and had increased to 325 ± 175 mL by *T*_5_. All other hemodynamic data are summarized in Table 1. Preload, as indicated by GEDI, decreased significantly after each phase in the hypovolemic group compared to baseline and dropped by 36% of its baseline value by the end of the experiment. The change of the other macrohemodynamic variables followed a similar pattern. When comparing the sham versus hypovolemic animals, variables differed between the two groups from *T*_1_, but significant differences over time continued only for GEDI, CVP and SVI. In the sham group there were no significant changes over time throughout the experiment.

### 3.2. Effects on Oxygen Balance

Variables related to oxygen balance are listed in Table 2. In the hypovolemic group DO_2_ fell significantly from *T*_1_ and remained so for the rest of the experiment. The VO_2_/DO_2_ increased significantly over 30% from *T*_1_, while ScvO_2_ and CO_2_ gap followed this change only after *T*_2_. Lactate changed significantly from *T*_3_. There were no significant changes in the sham group throughout the experiment.

**Figure 1:**
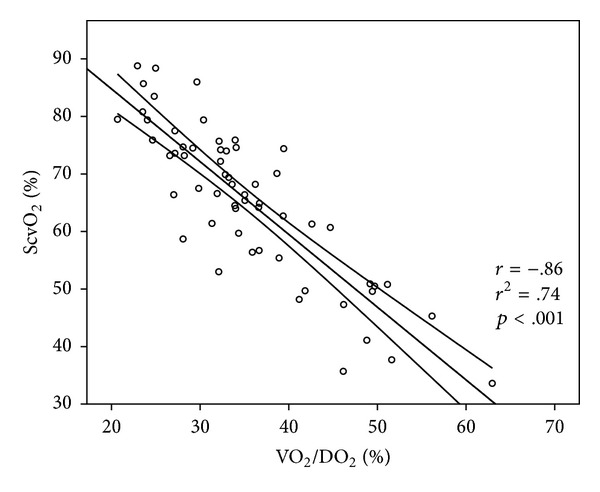
Correlation between VO_2_/DO_2_ and ScvO_2_. Data are presented as scatter with a linear regression line and its mean 95% confidence interval. VO_2_/DO_2_: oxygen extraction, ScvO_2_: central venous oxygen saturation.

**Figure 2:**
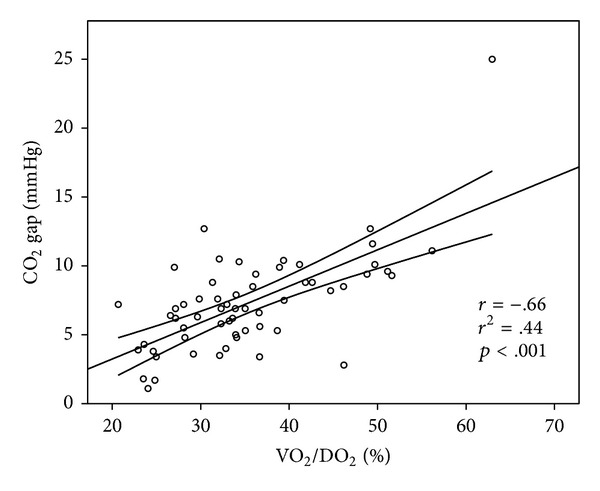
Correlation between VO_2_/DO_2_ and CO_2_ gap. Data are presented as scatter with a linear regression line and its mean 95% confidence interval. VO_2_/DO_2_: oxygen extraction; CO_2_ gap: central venous-to-arterial carbon dioxide difference.

In the hypovolemic group there was a significant correlation between VO_2_/DO_2_ and ScvO_2_ and CO_2_ gap (Figures 1 and 2). Lactate also showed a significant, but weak correlation with VO_2_/DO_2_ (*r* = .38, *r* ^2^ = .14; *p* < .05).

With receiver-operator characteristic (ROC) curves for ScvO_2_, CO_2_ gap and lactate to detect a VO_2_/DO_2_ > 30%, the area under the curves (AUC) was significant for ScvO_2_, CO_2_ gap (AUC ± SE = 0.887 ± 0.046; 0.783 ± 0.062; *p* < .05, resp.) while lactate did not reach statistical significance. The cut-off values to give the best sensitivity and specificity for ScvO_2_ and CO_2_ gap were 73% and 6.5 mmHg, respectively. Sensitivity, specificity, positive predictive, and negative predictive values for ScvO_2_ and CO_2_ gap are summarized in Table 3. Taking ScvO_2_ and CO_2_ gap values together to predict a VO_2_/DO_2_ > 30%, the false positive and false negative values were reduced.

### 3.3. Effects on Microcirculation

Variables related to microcirculation are listed in Table 4. In the hypovolemic group tonometry increased significantly only when measured in the intestines. Capillary perfusion rate and red blood cell velocity gradually and significantly decreased over time. This change was accompanied by a significant increase in BigET levels. In contrast, the sham group BigET decreased significantly by *T*_5_, while the other parameters remained unchanged. There was a significant difference in capillary perfusion rate, red blood cell velocity and BigET between the two groups.

The ROC curves for predicting VO_2_/DO_2_>30% proved to be significant for capillary perfusion rate and red blood cell velocity in the hypovolemic group. The area under curve did not differ significantly between these two parameters (AUC ± SE = 0.848 ± 0.084; 0.848 ± 0.092; *p* < .05, resp.).

The correlation between ScvO_2_ and the microcirculatory parameters proved to be significant apart from Ga-PCO_2_ (ScvO_2_-Ba-PCO_2_, -CPR, -RBCV, -BigET: *r* = −.38, *r* ^2^ = .15; *r* = .49, *r* ^2^ = .24; *r* = .40, *r* ^2^ = .16; *r* = −.47, *r* ^2^ = .23; *p* < .05, resp.). CO_2_ gap showed significant correlations with Ba-PCO_2_ and CPR (*r* = .48, *r* ^2^ = .23; *r* = −.51, *r* ^2^ = .26; *p* < .05, resp.).

## 4. Discussion

The main finding of our study is that it provides further evidence that low or decreasing ScvO_2_, as well as high or increasing CO_2_-gap can reflect changes and may be complementary in global oxygen balance and altered microcirculatory blood flow in hypovolemia.

### 4.1. Hemodynamic Changes

Our goal of achieving hypovolemia was reached as global end diastolic index, central venous pressure and stroke volume decreased significantly throughout the experiment, which resulted in a significant drop in cardiac index in the hypovolemia group. This decrease was notable until *T*_3_. Due to this change VO_2_/DO_2_ increased significantly from *T*_1_.

### 4.2. CO_2_ Gap and ScvO_2_ in Hypovolemia

Up until now, there has been consensus neither on the most accurate hemodynamic marker of hypovolemia nor on the endpoints for optimal fluid therapy [1, 16, 17]. Many recent studies have suggested that fluid therapy should be based on dynamic (such as cardiac output, pulse pressure variation and stroke volume variation) rather than static hemodynamic variables (such as CVP, pulmonary artery occlusion pressure), because they are better predictors of fluid responsiveness in ICU patients. However, pulse pressure variation and stroke volume variation are limited to patients who are fully ventilated and have no arrhythmias [18, 19]. Although it is not strictly a hemodynamic variable, in certain clinical conditions an ScvO_2_ value of ~70% has been used as a therapeutic endpoint to improve oxygen delivery [3, 7, 20, 21]. In a recent study it was found that a change in ScvO_2_ is a reliable parameter to define fluid responsiveness at the bedside in critically ill patients [5]. Similar results have been reported in two other studies demonstrating a close relationship between ScvO_2_ and cardiac index [6, 22]. However, one has to bear in mind that fluid resuscitation reduces hemoglobin levels, which may result in no change or decrease in ScvO_2_ therefore, the above may only hold true for hypovolemic patients with low ScvO_2_.

In our experiment the change in VO_2_/DO_2_, which increased significantly from *T*_1_, was accompanied by a fall in ScvO_2_, which is in accordance with previous findings. The change in VO_2_/DO_2_ was also accompanied by an increase in CO_2_ gap from *T*_2_. There is some evidence that CO_2_ gap increases in certain low flow states [8, 9]. The pathophysiology of increased CO_2_ gap may be due to the CO_2_ stagnation phenomenon. When cardiac output decreases, blood flow is slow and the washout is impaired; therefore, more CO_2_ is accumulated in the tissues, and as CO_2_ diffuses easily and quickly the CO_2_ gap increases [23].

ScvO_2_ showed very good sensitivity and specificity with a threshold of 73% for determining VO_2_/DO_2_ > 30%, which was further improved when CO_2_ gap >6.5 mmHg was added, leading to less false negative and false positive results. The ScvO_2_ and CO_2_ gap showed a significant and strong negative correlation. It is also important to note that lactate showed a significant but substantially weaker correlation to VO_2_/DO_2_ as compared to ScvO_2_ or CO_2_ gap. This highlights the limitation of lactate levels as therapeutic endpoint for resuscitation. This finding is in accordance with previously published data showing the limitation of lactate levels as therapeutic endpoint for resuscitation [24].

In cases when due to microcirculatory and/or mitochondrial defects oxygen uptake is insufficient, ScvO_2_ may be elevated (i.e., false negative). Previous studies have suggested that under such circumstances the increased value of CO_2_ gap (>5 mmHg) may help the clinician in detecting inadequate DO_2_ to tissues [8–10]. Our results lend further support to this theory. Furthermore, adding the CO_2_ gap to ScvO_2_ for identifying VO_2_/DO_2_ > 30%, there was an improvement in specificity, positive predictive, and negative predictive values.

We are not aware of any studies that have tailored hemodynamic support based on or supported by changes in CO_2_ gap; therefore, its clinical relevance remains unclear. However, our data clearly shows, and to our knowledge this is the first experiment to show that, an altered VO_2_/DO_2_ caused by hypovolemia is reflected by an increase in CO_2_ gap. Therefore its value may be an important alarm signal for the clinician and would help decision making at the bedside, especially when considering a fluid challenge or where commencing advanced hemodynamic monitoring is concerned.

### 4.3. Microcirculation

In any shock like states the microcirculation plays a vital role, as the most devastating effects of oxygen debt occur here in the cells [2, 22]. It has been demonstrated that microcirculatory disturbances can occur not only in cases of severe hypovolemic shock, but also in cases of a moderate hypovolemia without severe hypotension in human patients [25]. Recently, Bartels et al. evaluated the alteration of the sublingual microcirculation in response to controlled, central hypovolemia using sidestream dark field imaging in human subjects with intact autoregulation. They confirmed that despite adequate compensation of hypovolemia it can still be associated with decreased microcirculatory response, consequently with decreased oxygen delivery to the tissues [26].

In a prospective observational study in patients with septic shock it was found that the capillary perfusion rate was different in survivors (in whom it was increased) as compared to nonsurvivors. Moreover, it was the only factor to differentiate survivors and patients dying of multiple-system organ failure after the shock had resolved [2]. In accordance with these results we saw a gradual and significant decrease in both capillary perfusion rate and red blood cell velocity over time. There was also a very good area under curve when defining VO_2_/DO_2_ > 30% and good correlation with ScvO_2_ and CO_2_ gap. All these changes were observed only in the hypovolemic, but not in the sham group.

According to the microcirculatory parameters measured in this experiment, the inflicted hypovolemia resulted in significant changes to the microcirculation. Tonometry showed a significant increase in Ba-PCO_2_ due to hypovolemia, indicating decreased blood flow in the intestines. This is in accordance with previously published reports [27]. In contrast, there was no significant change in the Ga-PCO_2_. Regarding the importance and the value of gastric mucosal pH is controversial and its routine use has declined in intensive care over the last decades [28–30]. The difference between Ga-PCO_2_ and Ba-PCO_2_ is an interesting observation and also difficult to explain. However monitoring both has already been suggested, in order to give a small additional value in the diagnosis of possible mismatch in splanchnic perfusion [31, 32].

There was also a significant correlation between Ba-PCO_2_ both with ScvO_2_ and CO_2_ gap.

Little is known about BigET, but there is some evidence that ET-1 reflects tissue hypoxia [33]. However, in contrast to the insignificant change in BigET found in healthy volunteers suffering from acute hypoxia, our results showed a significant difference of BigET levels between the hypovolemic and the sham groups, which indicates that there is an effect of hypovolemia-induced tissue hypoxia on BigET levels [34].

### 4.4. Limitations of the Study

One of the possible limitations of our study is the long preparation period which might have resulted in the slightly elevated lactate levels although this was observed in both groups. Its clinical impact may be limited as a decreased ScvO_2_ and elevated CO_2_ gap may be influenced by several factors other than hypovolemia, including heart failure, severe sepsis/septic shock, and multiple trauma; thus, these results can only be applied when these conditions are unlikely to be present, for example, in postoperative critical care. Furthermore, these data were obtained in anesthetized animals and may not be the same in conscious human subjects. Finally, as there are no gold standards for hypovolemic animal experiments, therefore one cannot exclude that the choice of furosemide-caused hypovolemia may not be the most appropriate model. The disadvantage of this model is that it does not replicate real life clinical diseases. Traumatic hypovolemia and hypovolemia associated with sepsis are associated with profound microcirculatory changes which become superimposed on the changes following hypovolemia. This is particularly important in patients with sepsis, where ScvO_2_ is known to be a poor marker of tissue oxygenation. Indeed, in patients with sepsis, a high rather than a low ScvO_2_ is predictive of mortality.

## 5. Conclusion

Our results have shown that in addition to central venous oxygen saturation (ScvO_2_), central venous-to-arterial CO_2_ difference (CO_2_ gap) may also be used as a simple, but valuable indicator of hypovolemia-caused imbalance of oxygen extraction (VO_2_/DO_2_). Further clinical studies have to validate its clinical merits in indicating and tailoring hemodynamic support.

## Figures and Tables

**Table 1:** Hemodynamic changes.

		*T*_0_	*T*_1_	*T*_2_	*T*_3_	*T*_4_	*T*_5_
GEDI (mL/m^2^)	HG	349 ± 51	293 ± 53^∗‡#^	257 ± 47^∗‡#^	246 ± 53^∗#^	233 ± 42^∗#^	223 ± 31^∗#^
	SG	350 ± 26	387 ± 43	360 ± 22	365 ± 76	356 ± 51	349 ± 46
CVP (mmHg)	HG	7 ± 4	4 ± 3^∗‡#^	4 ± 3^∗#^	4 ± 3^∗‡#^	3 ± 3^∗#^	4 ± 3^∗#^
	SG	8 ± 1	7 ± 1	7 ± 1	7 ± 2	7 ± 1	7 ± 2
MAP (mmHg)	HG	121 ± 15	107 ± 17^∗‡^	94 ± 19^∗‡^	86 ± 15^∗‡^	85 ± 15*	83 ± 17*
	SG	120 ± 13	121 ± 14	117 ± 15	113 ± 16	121 ± 24	111 ± 25
HR (1/beats)	HG	78 ± 14	83 ± 15^∗‡^	93 ± 17*	102 ± 19*	130 ± 28^∗‡^	142 ± 28^∗‡#^
	SG	74 ± 12	75 ± 12	74 ± 9	78 ± 10	83 ± 16	80 ± 9
CI (L/min/m^2^)	HG	2.30 ± 0.35	1.78 ± 0.31^∗‡^	1.54 ± 0.32^∗‡^	1.46 ± 0.35^∗‡^	1.52 ± 0.41*	1.58 ± 0.36*
	SG	2.38 ± 0.50	2.65 ± 0.65	2.42 ± 0.48	2.38 ± 0.76	2.32 ± 0.50	2.27 ± 0.27
SVI (mL/m^2^)	HG	29 ± 5	19 ± 2^∗‡#^	15 ± 4^∗#^	14 ± 4^∗#^	12 ± 4^∗#^	12 ± 3^∗#^
	SG	33 ± 4	34 ± 6	33 ± 3	32 ± 6	31 ± 8	28 ± 5

GEDI: global end-diastolic volume index; CVP: central venous pressure; MAP: mean arterial pressure; HR: heart rate; CI: cardiac index; SVI: stroke volume index. *T*_0_: baseline measurement; *T*_1_–*T*_5_: five intervals. *p < .05 as compared to *T*_1_; ^‡^ *p* < .05 as compared to the previous value; RM ANOVA; ^#^ *p* < .05 HG versus SG; Mann-Whitney *U*-test with Bonferroni correction.

**Table 2:** Changes of oxygen balance.

		*T*_0_	*T*_1_	*T*_2_	*T*_3_	*T*_4_	*T*_5_
SaO_2_ (%)	HG	94 ± 6	95 ± 5	95 ± 2	96 ± 2	94 ± 3	94 ± 2
	SG	95 ± 5	94 ± 8	96 ± 5	94 ± 8	95 ± 5	94 ± 7
Hb (g/L)	HG	122 ± 9	129 ± 13	132 ± 7*	134 ± 7^∗‡^	137 ± 7^∗‡^	138 ± 8*
	SG	119 ± 10	115 ± 9	110 ± 6	108 ± 11	111 ± 9	102 ± 10
DO_2_I (mL/min/m^2^)	HG	361 ± 39	302 ± 52^∗‡^	268 ± 47^∗‡^	258 ± 55*	269 ± 65*	284 ± 55*
	SG	365 ± 78	396 ± 88	346 ± 73	337 ± 67	324 ± 55	298 ± 36
VO_2_/DO_2_ (%)	HG	28 ± 5	31 ± 6^∗‡^	34 ± 7^∗‡^	39 ± 9^∗‡^	41 ± 9*	40 ± 10*
	SG	25 ± 4	25 ± 6	26 ± 7	29 ± 5	27 ± 6	30 ± 7
ScvO_2_ (%)	HG	74 ± 10	71 ± 10	67 ± 11^∗‡^	64 ± 14*	59 ± 13^∗‡^	57 ± 14*
	SG	77 ± 8	76 ± 10	76 ± 9	75 ± 11	73 ± 14	73 ± 12
CO_2_-gap (mmHg)	HG	4.3 ± 2.3	7.5 ± 3.3	7.1 ± 2.6*	8.3 ± 2.8*	7.3 ± 2.9*	10.1 ± 5.5^∗#^
	SG	4.1 ± 2.4	3.5 ± 1.9	4.5 ± 1.3	3.9 ± 2.9	4.6 ± 1.9	4.4 ± 1.9
Lactate (mmol/L)	HG	3.8 ± 1.4	3.9 ± 1.3	4.3 ± 0.9	4.7 ± 0.9^∗‡^	5.1 ± 1.2^∗‡^	5.3 ± 1.5*
	SG	3.8 ± 0.9	3.9 ± 1.2	4.2 ± 1.8	4.6 ± 2.1	4.7 ± 2.7	4.9 ± 3.2
VO_2_I (mL/min/m^2^)	HG	98 ± 11	93 ± 16	88 ± 9	96 ± 7^‡^	104 ± 10^‡^	109 ± 16
	SG	88 ± 16	96 ± 6	85 ± 12	96 ± 8	85 ± 12	88 ± 14

SaO_2_: arterial oxygen saturation; Hb: hemoglobin; DO_2_: oxygen delivery; VO_2_/DO_2_: oxygen extraction ratio; ScvO_2_: central venous oxygen saturation; CO_2_ gap: venous-to-arterial carbon dioxide difference; VO_2_: oxygen consumption. *T*_0_: baseline measurement; *T*_1_–*T*_5_: five intervals. *p < .05 as compared to *T*_1_; ^‡^ *p* < .05 as compared to the previous value; RM ANOVA; ^#^ *p* < .05 HG versus SG; Mann-Whitney *U*-test with Bonferroni correction.

**Table 3:** Complementation of ScvO_2_ with CO_2_ gap.

	Sensitivity (%)	Specificity (%)	PPV (%)	NPV (%)
ScvO_2 _ ≤ 73%	78	83	91	63
CO_2_ gap > 6 mm Hg	71	72	85	52
ScvO_2_ + CO_2_ gap (≤73%) (>6 mm Hg)	58	100	100	72

ScvO_2_: central venous oxygen saturation; CO_2_ gap: venous-to-arterial carbon dioxide difference; PPV: positive redictive value; NPV: negative predictive value.

**Table 4:** Changes in microcirculation.

		*T*_0_	*T*_1_	*T*_2_	*T*_3_	*T*_4_	*T*_5_
Ba-PCO_2_ (mmHg)	HG	24 ± 8	35 ± 16^∗‡^	35 ± 17	36 ± 13*	33 ± 13*	37 ± 16*
	SG	19 ± 5	22 ± 9	20 ± 6	22 ± 11	22 ± 10	20 ± 8
Ga-PCO_2_ (mmHg)	HG	40 ± 12	43 ± 14	42 ± 14	39 ± 14	36 ± 10	37 ± 11
	SG	36 ± 22	37 ± 24	34 ± 21	32 ± 21	34 ± 20	32 ± 18
CPR (%)	HG	82 ± 15	—	53 ± 11^∗‡#^	—	—	45 ± 16^∗‡#^
	SG	91 ± 50		81 ± 80			83 ± 70
RBCV (*μ*m/s)	HG	887 ± 141	—	509 ± 120^∗‡#^	—	—	463 ± 209^∗#^
	SG	1054 ± 141		848 ± 194			963 ± 51
BigET (fmol/mL)	HG	1.44 ± 0.53	—	1.97 ± 0.84^∗‡^	—	—	2.29 ± 0.89^∗#^
	SG	1.36 ± 0.93		1.49 ± 1.27			0.98 ± 0.92*

Ba-PCO_2_: small bowel-to-arterial carbon dioxide difference; Ga-PCO_2_: gastric-to-arterial carbon dioxide difference; CPR: capillary perfusion rate; RBCV: red blood cell velocity; BigET: big endothelin. *T*_0_: baseline measurement; *T*_1_–*T*_5_: five intervals. *p < .05 as compared to *T*_1_, ^‡^ *p* < .05 as compared to the previous value, GLM repeated measures ANOVA; ^#^ *p* < .05 HG versus SG, Mann-Whitney *U*-test with Bonferroni correction.

## References

[1] Perner A (2009). Diagnosing hypovolemia in the critically ill. *Critical Care Medicine*.

[2] Sakr Y, Dubois MJ, De Backer D, Creteur J, Vincent JL (2004). Persistent-microcirculatory alterations are associated with organ failure and death in patients with septic shock. *Critical Care Medicine*.

[3] Rivers E, Nguyen B, Havstad S (2001). Early goal-directed therapy in the treatment of severe sepsis and septic shock. *The New England Journal of Medicine*.

[4] Sakr Y, Vincent J-L, Reinhart K (2005). High tidal volume and positive fluid balance are associated with worse outcome in acute lung injury. *Chest*.

[5] Giraud R, Siegenthaler N, Gayet-Ageron A, Combescure C, Romand J-A, Bendjelid K (2011). ScvO_2_ as a marker to define fluid responsiveness. *Journal of Trauma*.

[6] Krantz T, Warberg J, Secher NH (2005). Venous oxygen saturation during normovolaemic haemodilution in the pig. *Acta Anaesthesiologica Scandinavica*.

[7] Collaborative Study Group on Perioperative ScvO2 Monitoring (2006). Multicentre study on peri- and postoperative central venous oxygen saturation in high-risk surgical patients. *Critical Care*.

[8] Vallée F, Vallet B, Mathe O (2008). Central venous-to-arterial carbon dioxide difference: an additional target for goal-directed therapy in septic shock?. *Intensive Care Medicine*.

[9] Futier E, Robin E, Jabaudon M (2010). Central venous O_2_ saturation and venous-to-arterial CO_2_ difference as complementary tools for goal-directed therapy during high-risk surgery. *Critical Care*.

[10] Vallet B, Lebuffe G (2007). How to titrate vasopressors against fluid loading in septic shock. *Advances in Sepsis*.

[11] Groner W, Winkelman JW, Harris AG (1999). Orthogonal polarization spectral imaging: a new method for study of the microcirculation. *Nature Medicine*.

[12] Boda D, Kaszaki J, Tálosi G (2006). A new simple tool for tonometric determination of the PCO_2_ in the gastrointestinal tract: *in vitro* and *in vivo* validation studies. *European Journal of Anaesthesiology*.

[13] Kourembanas S, Marsden PA, McQuillan LP, Faller DV (1991). Hypoxia induces endothelin gene expression and secretion in cultured human endothelium. *Journal of Clinical Investigation*.

[14] Phillips CR, Vinecore K, Hagg DS (2009). Resuscitation of haemorrhagic shock with normal saline vs. lactated Ringer’s: effects on oxygenation, extravascular lung water and haemodynamics. *Critical Care*.

[15] Saugel B, Umgelter A, Schuster T, Phillip V, Schmid RM, Huber W (2010). Transpulmonary thermodilution using femoral indicator injection: a prospective trial in patients with a femoral and a jugular central venous catheter. *Critical Care*.

[16] Barros JMP, Do Nascimento P, Marinello JLP (2011). The effects of 6% hydroxyethyl starch-hypertonic saline in resuscitation of dogs with hemorrhagic shock. *Anesthesia and Analgesia*.

[17] Vallet B (2011). Intravascular volume expansion: which surrogate markers could help the clinician to assess improved tissue perfusion?. *Anesthesia and Analgesia*.

[18] Michard F, Teboul J-L (2002). Predicting fluid responsiveness in ICU patients: a critical analysis of the evidence. *Chest*.

[19] Monnet X, Osman D, Ridel C, Lamia B, Richard C, Teboul J-L (2009). Predicting volume responsiveness by using the end-expiratory occlusion in mechanically ventilated intensive care unit patients. *Critical Care Medicine*.

[20] Pearse R, Dawson D, Fawcett J, Rhodes A, Grounds RM, Bennett ED (2005). Changes in central venous saturation after major surgery, and association with outcome. *Critical Care*.

[21] Teixeira C, Da Silva NB, Savi A (2010). Central venous saturation is a predictor of reintubation in difficult-to-wean patients. *Critical Care Medicine*.

[22] Liakopoulos OJ, Ho JK, Yezbick A (2007). An experimental and clinical evaluation of a novel central venous catheter with integrated oximetry for pediatric patients undergoing cardiac surgery. *Anesthesia and Analgesia*.

[23] Vallet B, Teboul J-L, Cain S, Curtis S (2000). Venoarterial CO_2_ difference during regional ischemic or hypoxic hypoxia. *Journal of Applied Physiology*.

[24] Jansen TC, Van Bommel J, Bakker J (2009). Blood lactate monitoring in critically ill patients: a systematic health technology assessment. *Critical Care Medicine*.

[25] Ward KR, Tiba MH, Ryan KL (2010). Oxygen transport characterization of a human model of progressive hemorrhage. *Resuscitation*.

[26] Bartels SA, Bezemer R, Milstein DMJ (2011). The microcirculatory response to compensated hypovolemia in a lower body negative pressure model. *Microvascular Research*.

[27] Walley KR, Friesen BP, Humer MF, Phang PT (1998). Small bowel tonometry is more accurate than gastric tonometry in detecting gut ischemia. *Journal of Applied Physiology*.

[28] Creteur J, De Backer D, Vincent J-L (1999). Does gastric tonometry monitor splanchnic perfusion?. *Critical Care Medicine*.

[29] Palizas F, Dubin A, Regueira T (2009). Gastric tonometry versus cardiac index as resuscitation goals in septic shock: a multicenter, randomized, controlled trial. *Critical Care*.

[30] Steiner LA, Staender S, Sieber CC, Skarvan K (2007). Effects of simulated hypovolaemia on haemodynamics, left ventricular function, mesenteric blood flow and gastric PCO_2_. *Acta Anaesthesiologica Scandinavica*.

[31] Otte JA, Huisman AB, Geelkerken RH, Kolkman JJ (2008). Jejunal tonometry for the diagnosis of gastrointestinal ischemia. Feasibility, normal values and comparison of jejunal with gastric tonometry exercise testing. *European Journal of Gastroenterology and Hepatology*.

[32] Thorén A, Jakob SM, Pradl R, Elam M, Ricksten S-E, Takala J (2000). Jejunal and gastric mucosal perfusion versus splanchnic blood flow and metabolism: an observational study on postcardiac surgical patients. *Critical Care Medicine*.

[33] Lal H, Yu Q, Ivor Williams K, Woodward B (2000). Hypoxia augments conversion of big-endothelin-1 and endothelin ET_B_ receptor-mediated actions in rat lungs. *European Journal of Pharmacology*.

[34] Lenz T, Nadansky M, Gossmann J, Oremek G, Geiger H (1998). Exhaustive exercise-induced tissue hypoxia does not change endothelin and big endothelin plasma levels in normal volunteers. *American Journal of Hypertension*.

